# Cryoablation for the treatment of ventricular tachycardia in close proximity to coronary arteries

**DOI:** 10.1016/j.hrcr.2022.07.018

**Published:** 2022-08-06

**Authors:** Michael Ghannam, Aman Chugh, Michael Thomas, Jackson J. Liang, Frank Bogun

**Affiliations:** University of Michigan, Ann Arbor, Michigan

**Keywords:** Ventricular tachycardia, Catheter ablation, Cryoablation, Coronary angiography, Arrhythmogenic right ventricular cardiomyopathy

## Introduction

An otherwise healthy 37-year-old woman was referred for evaluation of palpitations, exercise intolerance, and near-syncope. Her episodes typically occurred during or shortly after exercising (she had been running regularly since high school). On 1 occasion, she measured her radial pulse rate at 244 beats per minute. Her presenting electrocardiogram showed normal sinus rhythm with T-wave inversion in leads V_1_-V_3_ (Supplemental Figure 1). Echocardiography showed normal left ventricular ejection fraction. An ambulatory patch monitor revealed a moderate number of monomorphic premature ventricular contractions (PVC; burden of 3.6%) and nonsustained ventricular tachycardia (VT) lasting 4–5 beats. An exercise treadmill test was performed where she attained 15.7 METs and was without evidence of ischemia or cardiac symptoms. Monomorphic PVCs were present throughout the study and she developed nonsustained VT during peak exercise and recovery, which matched her PVC morphology (Supplemental Figure 2). Her mother had been diagnosed with a left bundle branch block but there was not family history of unexplained syncope or sudden death.

**Key Teaching Points**

• Aspects of the right ventricular outflow tract lay in close proximity to the left anterior coronary artery system. Coronary angiography in multiple views prior to ablation should be considered.
• Cryoablation has several advantages over radiofrequency ablation in terms of minimizing the risk of injury to the coronary artery system; however, collateral damage may still occur and close monitoring during lesion application is critical.
• Cryoablation can be used to treat reentrant ventricular tachycardias requiring multiple applications over a large area. Further work is needed to determine the long-term applicability and efficacy of this approach.

A delayed enhancement cardiac magnetic resonance imaging scan was performed, which showed a mildly dilated left ventricle (end-diastolic volume index of 113 mL/m^2^, reference values 56–100 mL/m^2^) with an ejection fraction of 51%. The right ventricle (RV) was mildly dilated (end-diastolic volume index 125 mL/m^2^, reference volume 47–103 mL/m^2^) with regional akinesia in the RV outflow tract, with an ejection fraction of 47%. There was no evidence of late gadolinium enhancement.

Given recurrent, sustained palpitations with possible hemodynamic compromise, she was admitted to the hospital for an urgent electrophysiology study and ablation.

## Electrophysiology study and ablation

**Figure 1:**
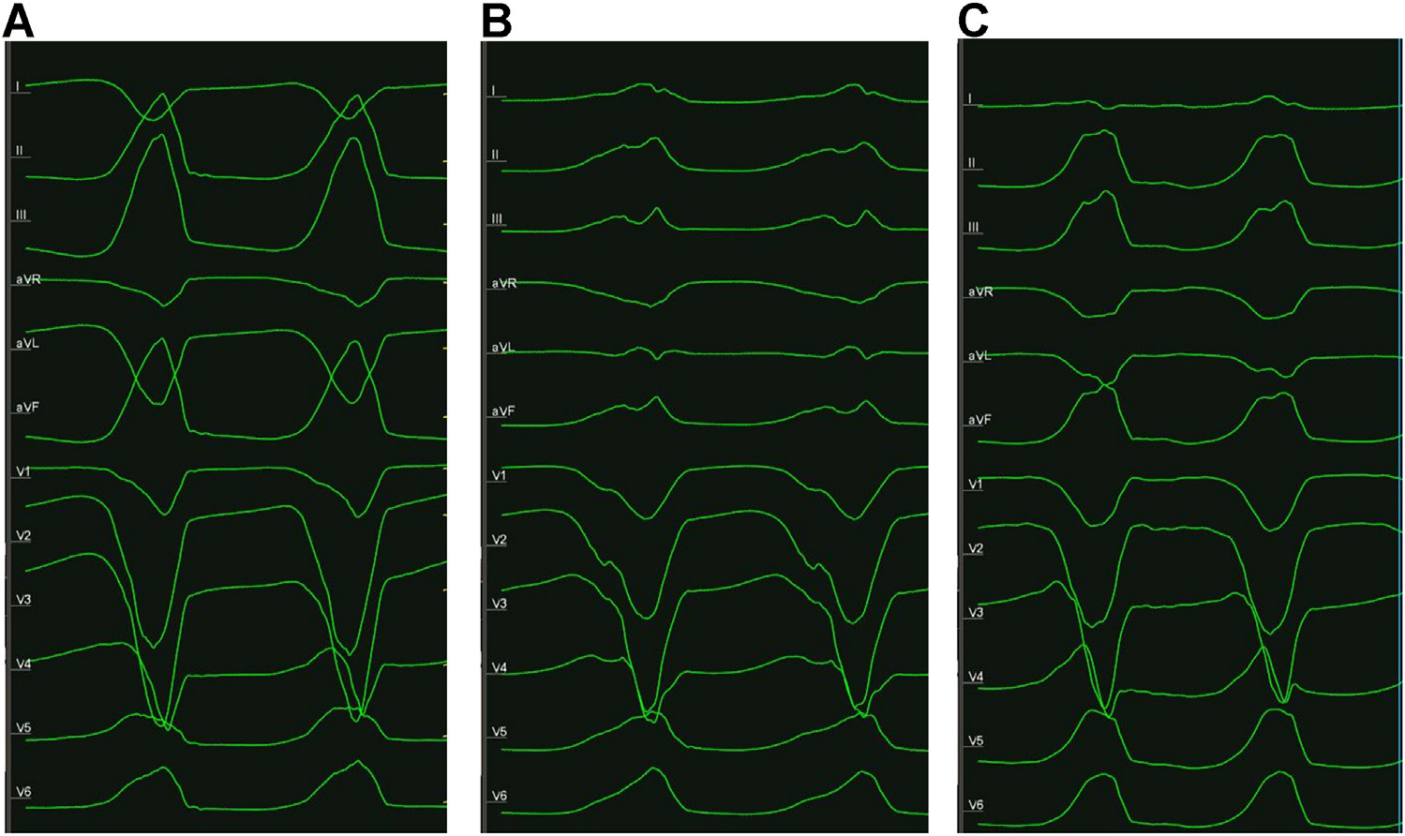
Induced ventricular tachycardia (VT). **A:** Initial programmed ventricular stimulation resulted in VT1. **B, C:** Two additional nonclinical VT morphologies were induced after initial targeting of VT1.

**Figure 2:**
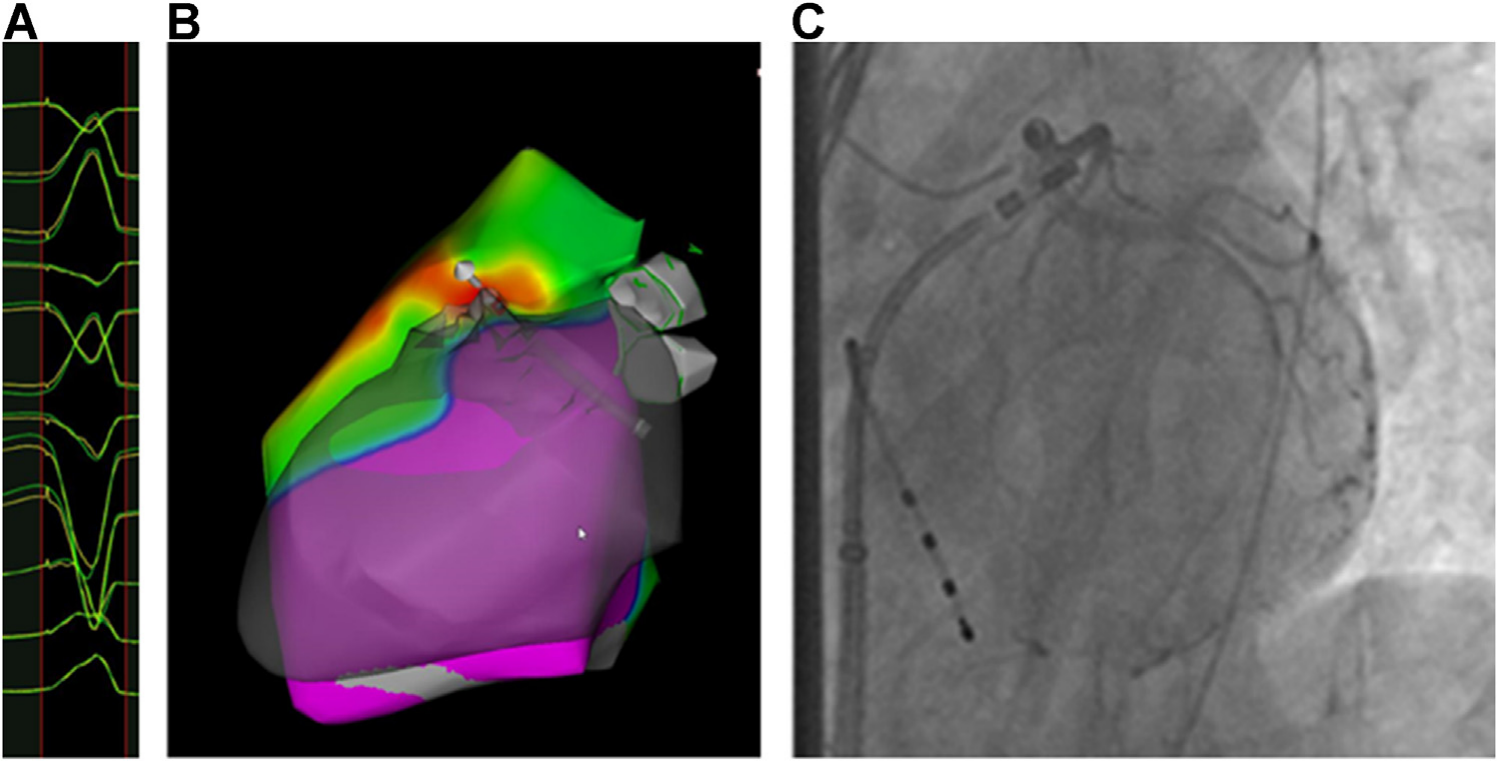
Electroanatomic mapping and coronary angiography. **A:** The clinical ventricular tachycardia (VT) was initially mapped to the septal right ventricular outflow tract where good pace maps (PASO module >95%) were attained. **B:** A correlation map to the clinical VT is shown; the catheter is positioned at the site with the best pace maps. **C:** Coronary angiography was performed prior to ablation, demonstrating the left anterior descending artery coursed very near (<1 mm) to the ablation area of interest.

The patient presented in normal sinus rhythm with infrequent PVCs matching her clinical morphology. With programmed ventricular stimulation (PVS) using triple extrastimuli, the patient developed a monomorphic VT with a left bundle branch block inferior axis at a cycle length of 290 ms (Figure 1A) and hypotension. Electroanatomic mapping was performed (CARTO; Biosense Webster, Diamond Bar, CA) using a 3.5 mm irrigated mapping and ablation catheter. Given hemodynamically unstable VT, pace mapping was performed (pacing at a cycle length 300 ms at 10 mV at 2.0 ms) beginning in the RV outflow tract (RVOT). Matching pace maps to the clinical VT were identified along the anteroseptal RVOT, with the best sites located in the anterior and septal RVOT (Figure 2A). These sites had abnormal electrograms and matched the clinical PVC as well (Supplemental Figure 3). Prior to radiofrequency (RF) energy delivery, coronary angiography was performed with the ablation catheter placed at the optimal target site (Figure 2B). Angiography performed in multiple planes revealed that the target site was in close proximity (<1 mm) to the course of the left anterior descending artery (Figure 2C and Supplemental Figure 4).

**Figure 3:**
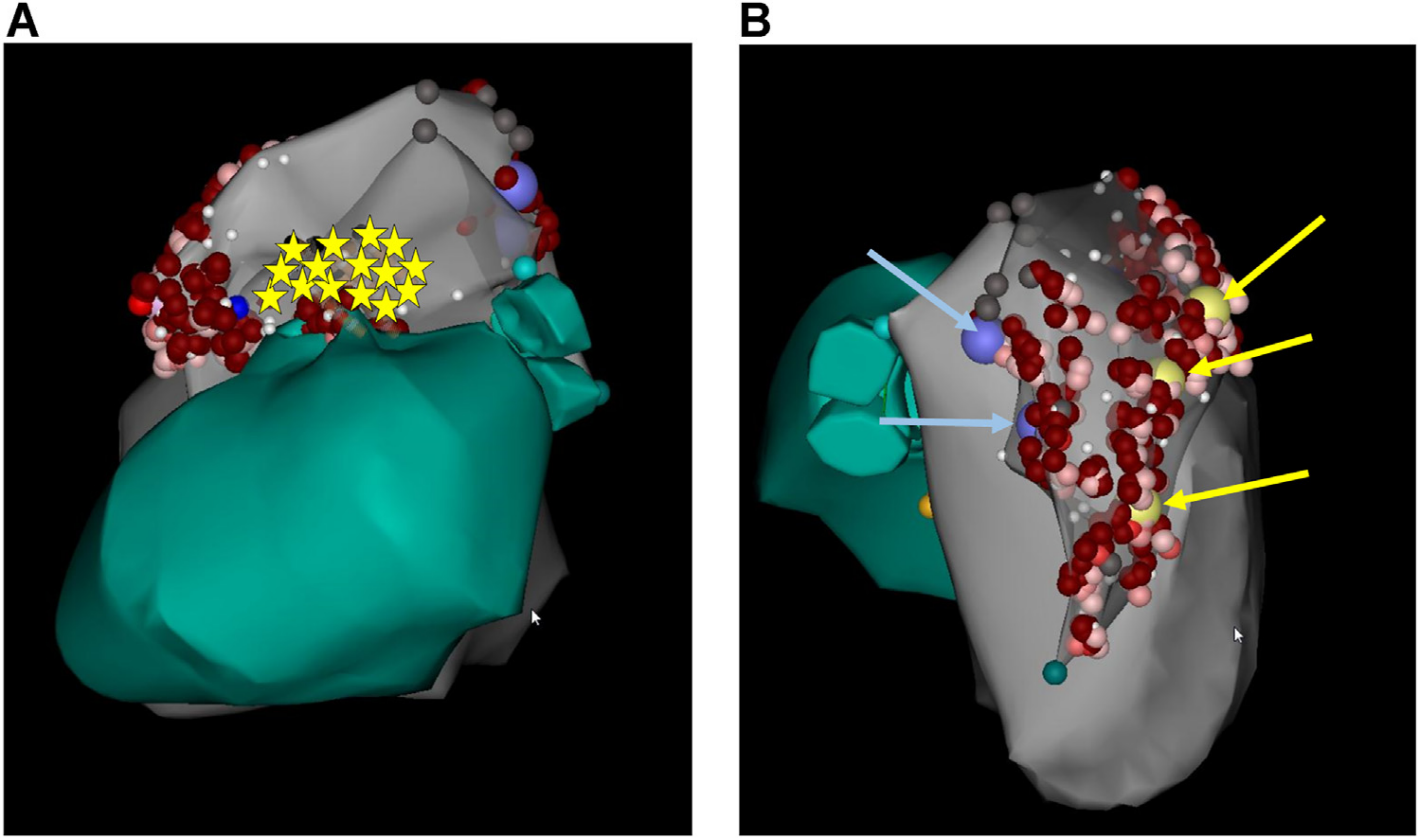
Anatomic contours and final ablation lesion set. **A:** Left lateral projection is shown. Yellow stars are representative of the cryocatheter lesions applied. Red markers are indicative of radiofrequency lesions. **B:** Posterior-anterior projection shown. Two additional nonclinical ventricular tachycardias were targeted, with the best pace maps at the large yellow and blue marks.

In order to minimize injury to the coronary artery, an 8 mm cryoablation catheter (Freezor MAX; Medtronic, Minneapolis, MN) was navigated to the target site. Cryoablation lesions were created by cooling the catheter tip to -80°C for 4 minutes, during which the 12-lead electrocardiogram was monitored closely for evidence of ischemia. In total, 20 cryo-lesions with complete freeze-thaw cycles were created throughout the septal RVOT at sites with matching pace maps (Figure 3A). Additional sites with abnormal voltage^1^ (displaying <10/12 pace maps to the clinical VT) located more inferiorly and apically along the septum were ablated with RF energy (25 W for up to 90 seconds targeting a 10 ohm impedance drop) after repeat angiography demonstrated an adequate distance between the ablation catheter and coronary arteries.

A repeat PVS was performed using the same protocol. The clinical VT was no longer inducible; however, 2 additional VTs were induced (Figure 1B and 1C), both with inferior and leftward axis with left bundle branch block morphologies. The additional VTs were ablated throughout the septum, anterior RV free wall, and posterolateral RV free wall (Figure 3). Subsequently, programmed ventricular stimulation was repeated, with and without isoproterenol infusion, and the patient was noninducible for any sustained arrhythmias, with the same protocol of programmed stimulation used prior to the ablation. Repeat coronary angiography confirmed patency of the coronary arteries (Supplemental Figure 5).

The patient elected for genetic testing, which was negative for pathologic variants. Her findings of T-wave inversions, inferiorly direct RVOT arrhythmias, and regional RV dyskinesia and dilation met task force criteria for arrhythmogenic right ventricular cardiomyopathy.^2^ Recommendation was made to abstain from intense exercise and to undergo implantation of a subcutaneous implantable cardiac defibrillator.^3^ Follow-up monitoring after 6 months has revealed no further ventricular arrhythmias.

## Discussion

This case demonstrates the safety and utility of combining cryoablation along with RF ablation to safely target reentry-related VT in the RVOT, where target sites may lie in proximity to the coronary artery system. Cryoablation has been described previously to target papillary muscle arrhythmias, para-hisian arrhythmias, and focal arrhythmias arising from the RVOT^4^; however, its utility in targeting reentrant VT, requiring ablation over a large area, has not been reported. Although entrainment mapping to confirm a reentry mechanism was not possible owing to hemodynamic instability, the patient had multiple VTs, each with matching pace maps at distinct sites, along with contiguous areas of abnormal voltage, and met diagnostic criteria for arrhythmogenic right ventricular cardiomyopathy, consistent with scar-based VT using broad isthmuses. Although RF ablation was also performed, only cryoablation was used in areas meeting criteria for target sites to the clinical VT, which was subsequently not seen on further PVS.

Coronary artery injury during ablation of ventricular arrhythmias is a rare but potentially catastrophic complication. Injury may be preceded by ST-segment changes or chest discomfort during ablation, but malignant arrhythmia or hemodynamic collapse may be the first manifestation. The importance of ensuring adequate distance to the coronary artery system prior to ablation in the epicardial space or coronary venous system is widely acknowledged; however, injury to the left anterior descending artery during RVOT ablation has also been reported.^5^ A comparative study using routine invasive and computed tomography angiography reported that aspects of the septal RVOT are an average of 2–4 mm of the left coronary artery system, well within the lesion depth possible with the use of irrigated ablation catheters.^6^ Thermal injury to the coronary arteries may result in various pathologic responses, including stenosis, vasospasm, or occlusion^7^; embolism from ablation catheter thrombus or char is also possible. Delayed presentation of coronary artery injury weeks after ablation has been reported, possibly owing to medial necrosis and intimal disruption.^8^

The optimal distance from the coronary arteries where RF ablation energy can be safely performed is unclear. A study of patients undergoing RF ablation within the coronary venous system reported that ablation within 2 mm of coronary arteries resulted in arterial damage in 50% of cases, while ablation >5 mm resulted in no instances of injury.^9^ A 5 mm cutoff has been suggested based on other animal and human studies.^10^ There is likely no single safety cutoff applicable to all circumstances owing to patient and procedural factors that affect vessels susceptible to injury. Larger-diameter arteries, high vessel flow rates, and the presence of neighboring intramural vessels create cooling effects, lowering the chance for thermal injury.^9^ Cardiac scar, irrigation fluid osmolarity, and ablation power parameters all impact lesion geometry are may result in more shallow lesions that spare deeper structures.^11^ In the present case, it is unclear if RF ablation could have been safely performed, though given the very close proximity (<1 mm) to the left coronary artery, it was not deemed appropriate despite the presence of matching VT target sites.

Cryoablation may present a safer alternative over RF ablation for lesions in close proximity to coronary arteries. Cryoablation requires long freeze cycles (2–4 minutes) to attain permanent tissue injury, which can be exploited to perform “cryomapping” where ablation can be interrupted if collateral tissue damage occurs.^12^ Cryo-lesions may also cause less arterial intimal damage and stenosis than RF ablation, even when performed in very close proximity (<2 mm) to coronary arteries.^13^ Caution is yet required, as acute and chronic arterial damage with cryoablation has been reported,^7^ with animal models showing neointimal proliferation as a possible mechanism of injury.^14^ Much of the established safety data of cryoablation with respect to coronary artery damage comes from its use during epicardial ablation^9^; however, this case demonstrates its potential use for endocardial ventricular ablation in close proximity to the coronary arteries as well.

While the use of cryoablation was important to the success of this procedure, further studies are needed to evaluate the widespread applicability of this approach. Particularly, the long-term success rate needs further evaluation. For example, focal cryoablation used for treatment of atrioventricular nodal reentrant tachycardia demonstrated similar acute success rate but higher arrhythmia recurrences when compared to RF ablation.^15^ A repeat exercise test may have provided additional prognostic information on her procedural outcome. Owing to the presence of inner lumina used for cooling fluid, cryocatheters are relatively stiff, with limited maneuverability, which may limit the ability to access critical endocardial sites. Lastly, because of the long freeze-thaw times and the need for multiple applications, this approach adds considerable procedural time.

## Conclusion

This case demonstrates the safety and efficacy of multiple focal cryoablation lesions for the treatment of reentry-based outflow tract arrhythmias requiring extensive ablation near coronary arteries. Prior to ablation within the RVOT, coronary angiography in multiple views can be performed to ensure an adequate distance from ablation sites to the coronary vasculature. Although no clear safety cutoff has been established, caution should be used for target sites within 5 mm of the coronary arteries and the use of cryoablation can be considered. Further work is needed to determine the long-term applicability and efficacy of this approach.

## AppendixSupplementary data

Suppelemental Material
