# Supplementary material for: Cryoablation for the treatment of ventricular tachycardia in close proximity to coronary arteries
Source: HeartRhythm Case Rep. 2022 Aug 6;8(10):707–10. doi: 10.1016/j.hrcr.2022.07.018 (PMC9596360; doi:10.1016/j.hrcr.2022.07.018)
Supplement: Suppelemental Material [file mmc1.docx]

Supplemental Figure 1


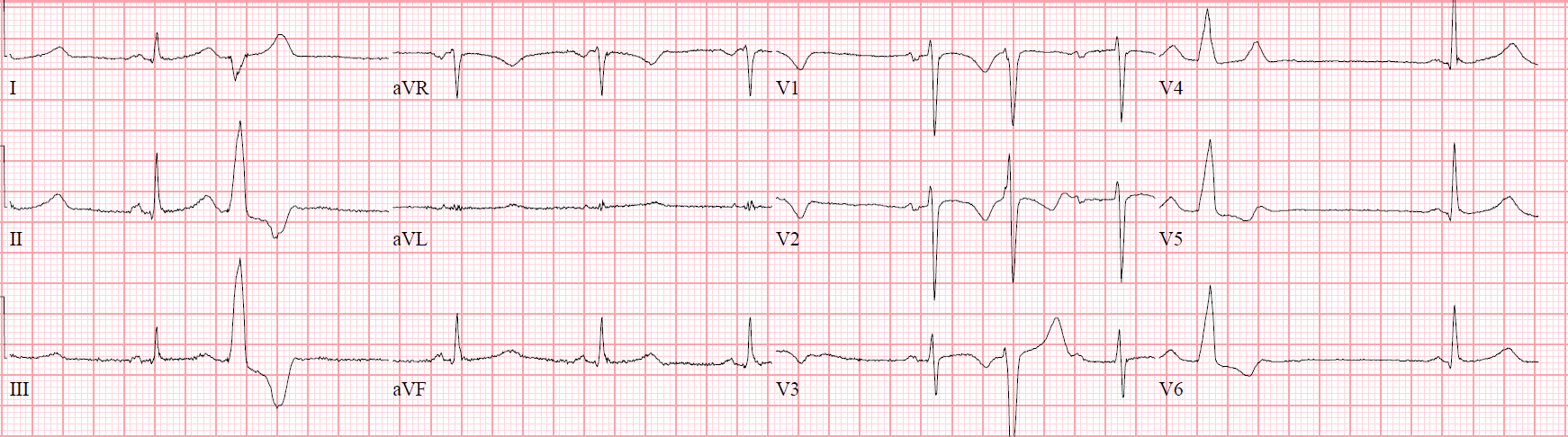


Supplemental Figure 1) **Presenting 12 lead EKG.** T-wave inversion in the right precordial leads are seen, an inferior axis left bundle branch premature ventricular contraction is present. Standard lead placement, sweep speed 25mm/s, 10mm/mV.

Supplemental Figure 2


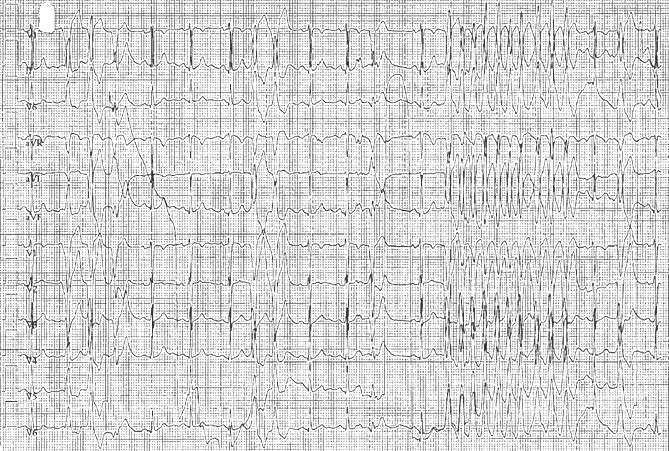


Supplemental Figure 2) **Exercise induced arrhythmia.** During peak exercise, the patient developed symptomatic premature ventricular contractions and non-sustained ventricular tachycardia matching her clinical morphology. Altered lead placement for exercise testing, sweep speed 25mm/s, 10mm/mV.

Supplemental Figure 3

**
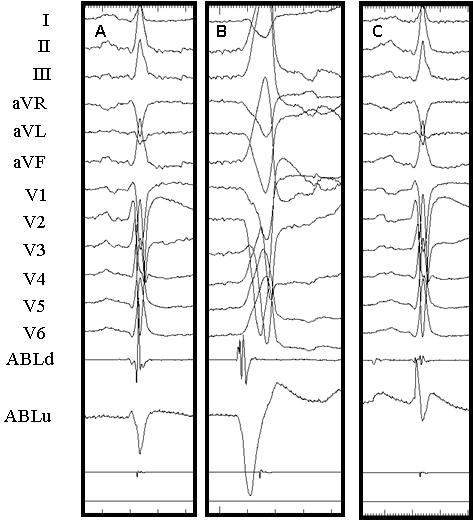
**

Supplemental Figure 3) **Effect of cryoablation on local electrograms.** **Panel A)** Pre-cryo lesion bipolar and unipolar electrograms at the site of matching pacemaps to the patient’s clinical VT. **Panel B)** A premature ventricular contraction matching the clinical VT is shown at the same catheter site prior to cyroablation. **Panel C)** A 240 second cryolesion was applied, with attenuation of the bipolar electrograms. After multiple lesion applications, non-capture was attained at the area of interest.

Supplemental Figure 4


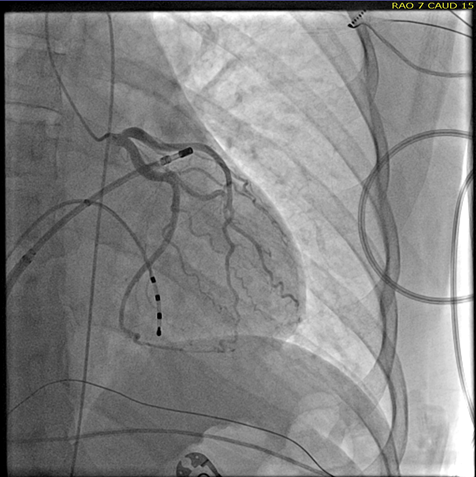


**A**

**B**

**C**


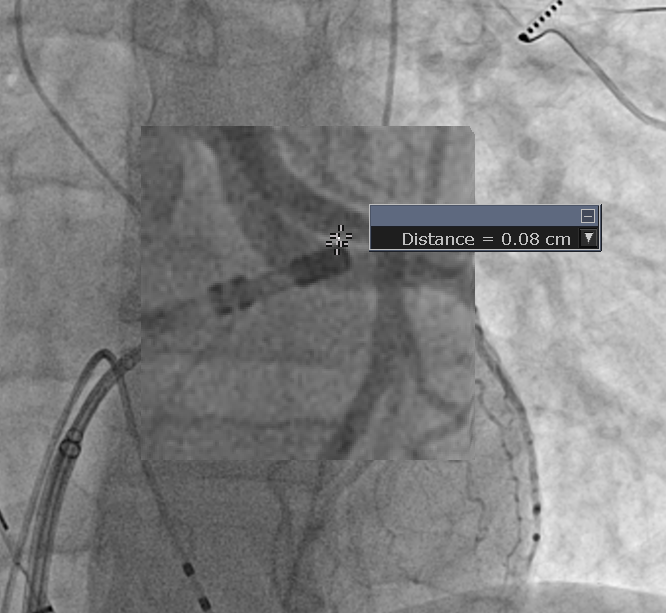


**D**

Supplemental Figure 4) **Coronary angiography.** Additional angiography in multiple angulations was performed prior to ablation at the area of interest, confirming proximity to the left anterior descending artery. **Panel A:** right anterior oblique 10 degrees, caudal 15 degrees. **Panel B:** left anterior oblique 43 degrees, caudal 20 degrees. **Panel C:** left anterior oblique 43 degrees, cranial 34 degrees. **Panel D:** Representative imaging showing measurements of the proximity between the ablation catheter and left anterior descending artery.

Supplemental Figure 5


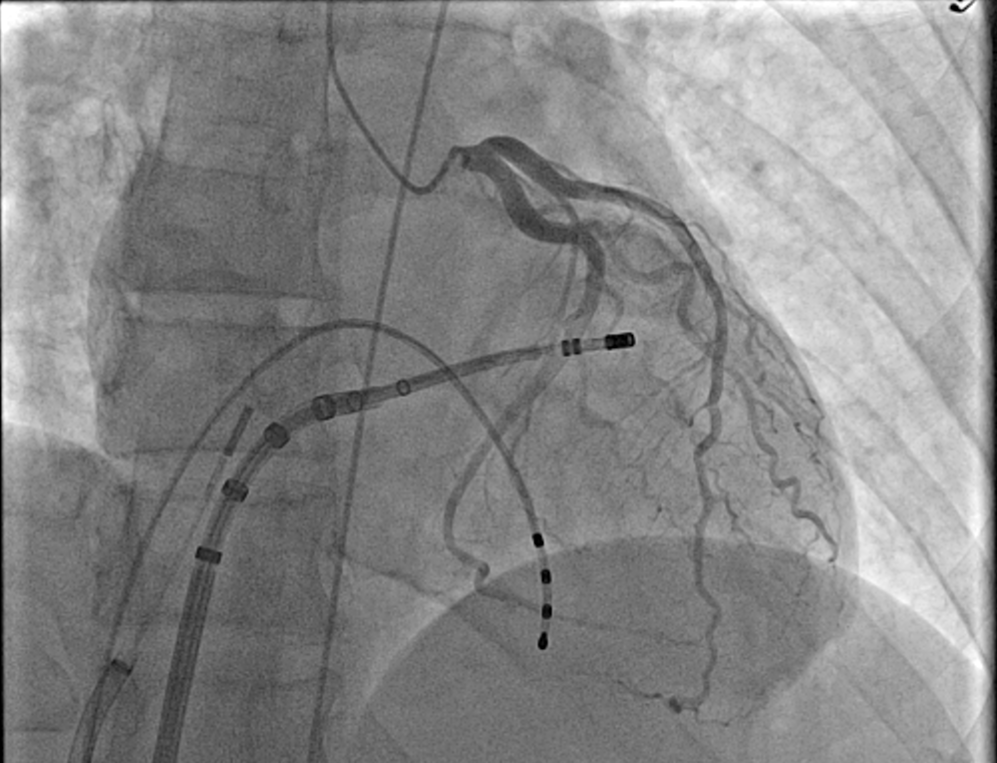


Supplemental Figure 5) **Post-ablation angiography.** After extensive cryoablation was performed in regions directly overlaying the left anterior descending artery, repeat angiography was performed confirming patency of this vessel. Projection is right anterior oblique 10 degrees, cranial 0 degrees.
